# Posttraumatic growth in young adults with parents diagnosed with cancer: Application of the self-regulation model

**DOI:** 10.1017/S1478951524001433

**Published:** 2024-11-08

**Authors:** Shiri Shinan-Altman, Liat Becker

**Affiliations:** 1 The Louis and Gabi Weisfeld School of Social Work, Bar-Ilan University, Ramat Gan, Israel; 2 Geha Mental Health Centre, Petakh Tikva, Israel

**Keywords:** Parental cancer, posttraumatic growth, illness representations, coping strategies, adult children

## Abstract

**Background:**

The rising incidence of cancer has led to an increased number of adult children impacted by parental cancer. Previous research primarily focused on younger individuals, leaving a gap in understanding the experiences of adult children aged 20–35.

**Objectives:**

To examine a model that integrates the interrelationships among the disease’s characteristics (i.e., disease stage), illness representations, coping strategies, and posttraumatic growth (PTG) in young adults with parents diagnosed with cancer. In addition, we examined indirect relationships involving illness representations as independent variables, coping strategies as mediators, and PTG as the outcome variable.

**Purpose:**

The rising incidence of cancer has led to an increased number of adult children impacted by parental cancer. Previous research primarily focused on younger individuals, leaving a gap in understanding the experiences of adult children aged 20–35. This study examines a model that integrates the interrelationships among the disease’s characteristics (i.e., disease stage), illness representations, coping strategies, and posttraumatic growth (PTG) in young adults with parents diagnosed with cancer. In addition, we examined indirect relationships involving illness representations as independent variables, coping strategies as mediators, and PTG as the outcome variable.

**Methods:**

A cross-sectional survey was conducted with 109 adult children (ages 20–35) of cancer patients. Data were collected using the Posttraumatic Growth Inventory, the Brief Illness Perception Questionnaire, and the COPE questionnaire. Path analysis was performed to test the study’s hypotheses.

**Results:**

The findings revealed that illness representations and coping strategies accounted for significant variance in PTG. Higher perceived severity of the parent’s illness was associated with greater use of problem-focused and emotion-focused coping strategies, which were linked to higher PTG. Lower perceived control over the illness was associated with less use of problem-focused coping and subsequently lower PTG.

**Conclusions:**

This study underscores the importance of subjective perceptions and coping strategies in fostering PTG among young adults with parents diagnosed with cancer. The findings highlight the need for tailored psychosocial interventions to enhance adaptive illness representations and effective coping strategies, promoting resilience and growth in this unique demographic.

## Background

The global incidence and burden of cancer have been rising steadily in the twenty-first century, making it the second leading cause of death (Siegel et al. 2024). Considering its annually increasing new case rates, the number of adult children impacted by parental cancer is also expected to rise (Landi et al. 2022; Morley et al. 2016).

Existing research on the impact of a parent’s cancer diagnosis on their children primarily centers on individuals aged 0–24 (Geertz et al. 2023; Ohan et al. 2020; Wuensch et al. 2022), leaving a gap in understanding the experiences of older adult children, particularly those between 19 and 35. In the current study, this research gap is addressed by focusing on adult children within this age range, which is identified as the stage of early adulthood (Erikson 1950). At this stage, adult children are grappling with the balance between forging personal relationships and assuming work-related responsibilities. Typically, they are seeking financial independence, independence from the parental home, and the establishment of intimate relationships (Maree 2021; Santilli et al. 2019). However, the presence of an ill parent in their lives introduces unique challenges. This cohort may experience high psychological distress associated with the parent’s diagnosis (i.e., the threat of death of a loved one) as well as with multiple role demands such as managing household tasks, coordinating medical appointments and care, and providing emotional support to the ill parent (He et al. 2022; Teixeira et al. 2016). In some cases, living with a sick parent may hinder career pursuits and economic independence. The resultant disruptions in these emerging adults’ developmental path may contribute to heightened distress (McDonald et al. 2016). However, along with the possible negative consequences of coping with a parent’s illness, there has also been growing interest in the positive dimensions among family members coping with a chronic illness of another family member, such as growth from a crisis (Nouzari et al. 2019; Palacio and Limonero 2020).

Posttraumatic growth (PTG) is defined as positive psychological changes experienced as a result of the struggle with highly stressful and challenging life events (Tedeschi and Calhoun 1996). These changes can manifest in various aspects of life, including an increased appreciation for life, spiritual growth, enhanced personal strength, improved relationships with others, and the discovery of new possibilities (Park et al. 1996; Tedeschi and Calhoun 2004). PTG challenges the traditional view that traumatic experiences necessarily result in negative outcomes and highlights the potential for growth and transformation (Wu et al. 2019). Recent studies focusing on PTG in the context of family illness, particularly among young adults, have found that adolescents and young adult with cancer and cancer survivors often exhibit significant PTG, which is closely linked to their levels of psychological resilience, particularly in the face of life-threatening illnesses (Atay Turan et al. 2023; Chen et al. 2020).

In the context of adult children who have a parent diagnosed with cancer, there is a growing awareness that these individuals may also experience PTG as they cope with their parent’s illness (Levesque and Maybery 2014; Morris et al. 2020; Teixeira and Pereira 2016). For example, in a study investigating predictive factors of positive outcomes among adult children (aged 18–70) of parents with cancer, it was revealed that greater levels of trauma experienced in response to the parent’s illness were significantly associated with more substantial PTG (Levesque and Maybery 2014). That said, although these studies (Levesque and Maybery 2014; Morris et al. 2020; Teixeira and Pereira 2016) have significant value, the study participants were described as being over the age of 18, but their particular developmental stage was not specified. Furthermore, although PTG is likely to involve numerous associated interacting factors (Henson et al. 2021), to our knowledge, no researchers to date have examined this construct among young adults with parents diagnosed with cancer guided by a theoretical framework. In order to overcome these shortcomings in the literature, in the current study we used the self-regulation model (SRM) (Leventhal et al. 1980) as a conceptual framework, and we specifically targeted individuals in the young adulthood.

The SRM offers a suitable framework for understanding PTG in young adults with parents diagnosed with cancer. The model proposes that outcomes (such as PTG) are shaped by individuals’ personal beliefs or representations of the illness, coping strategies, and perceptions of the consequences of the illness (Leventhal et al. 1980). Within this model, individuals formulate cognitive and emotional perceptions of health threats. Cognitive representations of a disease encompass 7 key categories: identity (the disease’s name and symptoms); causes (beliefs about its origins); timeline (its duration and progression); consequences (impact on life); control (effectiveness of actions or treatment in managing symptoms); illness coherence (understanding the disease); and cyclical timeline (its fluctuating nature). Additionally, an emotional component of the model assesses the emotional responses and impact of the disease, integrating these perceptions into a holistic understanding of how individuals relate to the illness of a loved one.

Previous studies examined PTG in the context of the SRM (Du et al. 2024; Fernández et al. 2023). For example, illness uncertainty and perceived social support mediated the relationship between PTG and fear of progression in brain tumor patients, showing how SRM-related illness perceptions influence psychological outcomes (Du et al. 2024). Another study found that while resilience and dispositional optimism are linked to adaptation in breast cancer patients, they did not significantly relate to PTG, highlighting the role of the SRM in understanding PTG (Fernández et al. 2023).

According to the SRM, illness representations are closely linked to coping, with coping serving as a mediator between illness representations and health outcomes (Hagger and Orbell 2022). Coping refers to cognitive and behavioral responses to stress, categorized into problem-focused and emotion-focused coping. Problem-focused coping involves strategies aimed at directly addressing and altering the stressor, such as problem-solving, time management, and information gathering. Emotion-focused coping, on the other hand, focuses on managing emotional responses to stress through techniques like seeking social support, positive reappraisal, and relaxation practices. Together, these coping mechanisms help individuals either change the stress-inducing circumstances or mitigate their emotional impact, enhancing overall stress management (Lazarus and Folkman 1984). The mediation of coping strategies in the link between illness representations and health outcomes has been examined in a few studies, but the majority of these investigations were carried out in populations grappling with their own chronic diseases, and in any event the results regarding the mediating impact of coping have been inconsistent (Shinan-Altman and Afuta-Goldstein 2020; Shinan-Altman and Katzav 2021; Tonapa et al. 2022).

Regarding the association between coping strategies and PTG, this relationship has been examined in several studies (e.g., Eissenstat et al. 2022; Morris et al. 2020). In a meta-analysis including 96 studies regarding the association between coping strategies and PTG (among other things), PTG was shown to have different correlations with 4 groups of coping strategies: problem-focused coping, positive emotion-focused coping, negative emotion-focused coping, and unclassified coping (Eissenstat et al. 2022). Additionally, in a literature review of studies that addressed psychosocial factors related to PTG among breast cancer survivors, it was found that coping strategies such as those that focused on solving problems, planning, and turning to religion had a distinct positive relationship with PTG (Kolokotroni et al. 2014). However, the association of coping strategies with PTG in the presence of clinical variables (e.g., disease stage) and illness representations specifically among young adults with parents diagnosed with cancer has, to date, not been examined.

### The current study

**Figure 1.**
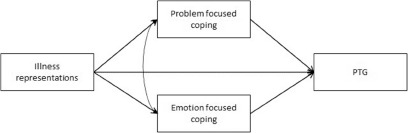
The study model for PTG, with illness representations and coping strategies.

Establishing objective (the disease’s characteristics) and subjective (illness representations, coping strategies) predictors of PTG in young adults with parents diagnosed with cancer is important for theoretical as well as practical reasons. Namely, establishing such predictors may enhance the construction of more elaborate models regarding the PTG of young adults with parents diagnosed with cancer which, in turn, may promote the design of more informed and effective psychosocial intervention programs aimed at promoting this cohort’s PTG. Based on the SRM, the aim of the present study was to examine a model that integrates the interrelationships among the disease’s characteristics (i.e., disease stage), illness representations, coping strategies, and PTG in young adults with parents diagnosed with cancer. In addition, we examined indirect relationships involving illness representations as independent variables, coping strategies as mediators, and PTG as the outcome variable. Figure 1 illustrates the study’s model.

*We hypothesized the following*:

H1. Illness representations and coping strategies would be positively associated with PTG.

H2. Coping strategies would mediate the relationships between illness representations and PTG, such that higher illness representations would be associated with greater use of the coping strategies, which in turn would be associated with higher PTG.

## Method

### Procedure

Questionnaires were administered through the Qualtrics online platform (www.qualtrics.com). Participants were invited via the Internet to take part in a survey on PTG of young adults with parents diagnosed with cancer. They were recruited mainly through Internet forums and websites dealing with cancer, as well as through social media outlets (Facebook pages focusing on cancer). In addition, participants were recruited via cancer associations. Notes on these websites contained a short explanation about the study and a link to the questionnaire. Ethics approval was received from the Ethics Committee of the XXX University in accordance with the Declaration of Helsinki (approval no. 11902). Participants indicated on the questionnaire form that they consent to participate in the study. Inclusion criteria were: (1) adult children between the ages of 20–35 who had a parent diagnosed with cancer; and (2) Hebrew speakers. Exclusion criteria were: (1) children of a parent diagnosed with cancer under the age of 20 or over the age of 35 (*n* = 3); and (2) children of a parent diagnosed with cancer who had already died as a result of the disease (*n* = 2). Sample size was determined using G*Power (Faul et al. 2009). For a multiple regression analysis with 7 predictors, a moderate effect size *f*^2^ = .15 (equals *R*^2^ = .13), *α* = .05, and power = .80, the required sample size is *N* = 103 participants.

### Measures

#### Dependent variable

The *Posttraumatic Growth Inventory* (Tedeschi and Calhoun 1996) was used to evaluate positive changes that individuals experience after a stressful event. Participants were asked to rate 22 items regarding the extent to which they experienced changes resulting from their parent’s disease on a Likert-type scale ranging from 0 (*no change*) to 5 (*significant change*). A sample item was, “I have a stronger sense of closeness to others.” A mean score was calculated, with higher scores indicating greater PTG. The internal consistency of the index was *α* = .96.

#### Independent variables

*Illness representations* were assessed using the Brief Illness Perception Questionnaire (Broadbent et al. 2006), which measures illness perception components as conceptualized by the SRM (Leventhal et al. 1980). The components included a single item for each of the following 8 dimensions, as applied to the study participants: the adult children’s perceptions of the consequences of the disease in relation to various domains in their lives; the timeline of the disease (chronic versus acute); self-control (the extent to which they can exert control over the disease); symptom burden on the adult child; perceived treatment control; concern regarding the disease; illness emotional representations (extent to which the illness makes them angry or fearful); and understanding of what the parent’s disease entails. A sample item was, “To what extent does your parent’s illness affect you emotionally?” Participants rated each dimension on a Likert-type scale ranging from 0 to 10, with a higher score representing more negative perceptions. Total Cronbach’s *α* was found to be low (.59) and thus a principal components factor analysis was calculated for the 8 items, using the criterion of Eigenvalue greater than 1, and oblique rotation. Three factors emerged: (1) severity of the effects on the adult child (4 items, Eigenvalue = 2.85, 35.63% of the variance, loadings .70–.85, *α* = .82); (2) self-control and perceived treatment control (2 items, Eigenvalue = 1.35, 16.81% of the variance, loadings .66 and .79, *r* = .26, *p* = .006), and (3) timeline and understanding of what the disease entailed (2 items, Eigenvalue = 1.10, 13.75% of the variance, loadings .58 and −.74, *r* = −.13, *p* = .197). The first 2 factors were defined with item means, and the third factor was not defined due to the negative correlation between the 2 items.

In order to assess perceived causes of the disease, participants were also asked to list up to 3 factors which they perceived to have been causes of their parent’s disease. Four sub-dimensions emerged: psychological attributes (e.g., “stress or worry”); risk factors (e.g., heredity, diet); immunity (e.g., “a germ or virus”); and accident or chance (e.g., “fate or bad luck”).

*Coping* strategies were assessed using the COPE, a 30-item Hebrew-language version of the questionnaire, based on the scale developed by Carver and colleagues (Carver et al. 1989). Coping strategies were grouped into 2 categories: problem-focused coping (15 items) and emotion-focused coping (15 items). A sample item was, “I try to get advice from someone about what to do.” Responses were rated on a Likert-type scale with 5 levels, ranging from 1 (*not at all*) to 5 (*very much*). An index score was calculated for each type of coping strategy by adding up the responses, with higher scores indicating greater use of that particular coping strategy. The internal consistency of the index was *α* = .85 for problem-focused coping and *α* = .66 for emotion-focused coping.

*Sociodemographic details* included age, gender (female/male), marital status (married/not married), place of birth (Israel/other), number of children, education, income (above average/average/below average income in Israel, as published by the Israel Central Bureau of Statistics), residential status (with parents/not with parents), age when the parent’s cancer was diagnosed, stage of the disease when it was diagnosed.

### Statistical analyses

Data were analyzed using SPSS ver. 28. Descriptive statistics were used to describe the participants’ demographic characteristics and the research variables. Internal consistencies were calculated for the study variables with Cronbach’s *α*. Pearson correlations were calculated to assess the associations between the research variables. Pearson correlations and *t*-tests were calculated for the background characteristics with the study variables in order to identify the background characteristics that should be controlled for when assessing the research model. The research model was assessed with a calculation of a path analysis, using AMOS ver. 28. Model fit was evaluated using *χ*^2^, normed fit index (*NFI*), non-normed fit index (*NNFI*), comparative fit index (*CFI*), and root mean square error of approximation (*RMSEA*). Mediation was examined within the path analysis, with bootstrapping of 5,000 samples and a bias-corrected 95% confidence interval. The Monte Carlo Method (Hayes 2022) for assessing mediation was used, and variables were standardized. The significance level was set at *p* < .05.

## Results

The study population included 109 adult children (ages 20–35) of a parent diagnosed with cancer. As shown in Table 1, the mean age was 32.90 years, with a range of 20–35. The majority of the participants were Israeli-born, with women making up the larger portion. About half were married, and the average number of children was 1.65. Most had a college/university education, and about half perceived their income to be above average. Most did not live with their parents, and their mean age when the parent’s cancer was diagnosed was about 26 years. At the time of diagnosis, 15% of cases were at Stage I, 23% at Stage II, 26% at Stage III, and 36% at Stage IV.

**Table 1.** Participants’ characteristics (*N* = 109)

*Sociodemographic characteristics*	
Mean age (SD), range	30.90 (4.99), 20–35
Gender (%)	
Male	26 (23.9)
Female	83 (76.1)
Marital status (%)	
Married	55 (50.5)
Not married	54 (49.5)
Place of birth (%)	
Israel	102 (94.4)
Other	6 (5.6)
Mean number of children (SD), range	1.65 (1.29), 0–6
Education (%)	
High school	23 (21.1)
College/university	86 (78.9)
Income (%)	
Above average	52 (49.5)
Average	10 (9.5)
Below average	43 (41.5)
Residential status (%)	
With parents	13 (11.9)
Not with parents	96 (88.1)
Mean age when the parent’s cancer was diagnosed (SD), range	26.02 (5.87), 9–36
Stage of the disease when diagnosed (%)	
Stage I	15 (15%)
Stage II	23 (23%)
Stage III	26 (26%)
Stage IV	36 (36%)

SD = standard deviation.

Table 2 presents means, SDs, ranges, and correlations for the study variables. As can be seen, the mean PTG score was moderate, and the parent’s cancer was perceived as having rather severe effects on the adult child, with rather low self-control and treatment control. Problem-focused coping was moderate, and emotion-focused coping was moderate-low. Disease stage was positively associated with the perceived severity of the effects of the parent’s cancer on the adult child, as well as with low self-control and treatment control. Further, perceived severity of the effects of the parent’s cancer on the adult child was positively associated with both problem-focused and emotion-focused coping, as well as with PTG. In addition, problem-focused and emotion-focused coping were positively associated with PTG. Low self-control and treatment control were unrelated to the coping strategies or to PTG.

**Table 2.** Means, SDs, ranges, and Pearson correlations for the study variables (*N* = 109)

Variables	1	2	3	4	5	6
1. PTG	–					
2.Stage of disease	−.06	–				
3. Severity of effects on adult child	.40***	.36***	–			
4. Low self-control and treatment control	.02	.38***	.12	–		
5. Problem-focused coping	.50***	.07	.25**	−.13	–	
6. Emotion-focused coping	.39***	−.01	.36***	.01	.07	–
Mean	2.75	2.83	7.29	7.11	3.14	2.51
SD	1.19	1.08	2.04	2.06	.68	.51
Possible range	0–5	1–4	0–10	0–10	1–5	1–5
Actual range	0–4.91	1–4	1.5–10	2–10	1.33–4.53	1.27–3.73

* *p* < .05, **p < .01, ***p < .001. Stage of the disease – Spearman correlations, SD = standard deviation.

Regarding the causes for their parent’s cancer as perceived by the participants, 76 participants responded (69.7%) to this item, and most noted at least 2 reasons. Fifty-one (67.1%) attributed the disease to psychological reasons; 58 (76.3%) to risk factors; 7 (9.2%) to immunity; and 15 (19.7%) to accident or chance.

In order to identify background variables that should be controlled for when examining the study hypotheses, the differences and associations between the background variables and the study variables were examined. First, PTG was found to be unrelated to disease stage, as were coping strategies (Table 2). Disease stage was positively associated with perceived severity of effects on the adult child and perceived low self-control and treatment control (Table 2). Examining these relationships with an analysis of variance revealed that the major difference was between Stages I and II vs. Stages III and IV (perceived severity of effects: Mean = 6.48, SD = 1.98 vs. Mean = 7.87, SD = 1.88, and perceived low control: Mean = 6.28, SD = 1.69 vs. Mean = 7.71, SD = 2.12, *p* < .001). Thus, disease stage was used dichotomously (I + II vs. III + IV) in subsequent analyses.

Gender was found to be significant for PTG and emotion-focused coping. PTG was found to be higher for women (Mean = 3.33, SD = 1.17) than men (Mean = 2.57, SD = 1.15), *t*(107) = 2.91, *p* = .004, as was emotion-focused coping (women: Mean = 2.72, SD = .44, men: Mean = 2.44, SD = .51), *t*(107) = 2.55, *p* = .012. Age was found to be significant for perceived severity of the effects (*r* = .20, *p* = .041), such that older participants perceived greater severity. Other background variables (marital status, having children, education, income, and residential status) were unrelated to the study variables. Thus, subsequent analyses included gender (1 – male, 0 – female), age, and disease stage (1 – Stages III + IV, 0 – Stages I + II) as control variables.

**Figure 2.**
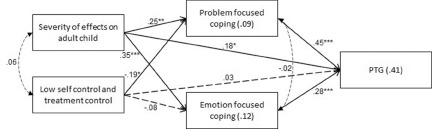
Path analysis for PTG, with illness representations and the coping strategies.

The study model was examined with a path analysis. Gender, age, and disease stage were controlled for, and were allowed to correlate among themselves. The 2 variables of illness representations (severity of effects on the adult child and low self-control and treatment control) served as the independent variables (with a correlation between them), and the coping strategies served as the mediators (with a correlation between them as well). PTG was the dependent variable. For the sake of clarity, the control variables are not presented in the figure. The model was found to fit the data: *χ*^2^(12) = 14.11, *p* = .294, *NFI* = .910, *NNFI* = .962, *CFI* = .984, *RMSEA* = .041. Figure 2 presents the results of the path analysis.

*R*^2^ – values within rectangles. *β* – standardized regression coefficients – values above 1-way arrows. Pearson *r* – correlations – values by 2-sided arrows. Solid arrows – significant associations, dashed arrows – non-significant associations.*p < .05, **p < .01, ***p < .001.

Results show that 41% of the variance in PTG was explained by the study variables, and illness representations explained 9% and 12% of the variance in problem-focused coping and emotion-focused coping, respectively. As shown in the figure, perceived higher severity of effects on the adult child was positively associated with both coping strategies, as well as with PTG. Perceived low self-control and perceived treatment control were negatively associated with problem-focused coping, such that greater self-control and treatment control were associated with a greater use of problem-focused coping. Both coping strategies were positively associated with PTG.

The 2 total indirect effects were significant: the total indirect effect between perceived severity of effects on the adult child and PTG (effect = .25, SE = .07, *p* < .001, 95% CI = .13, .42), and the total indirect effect between perceived low self-control and treatment control and PTG (effect = −.13, SE = .06, *p* = .033, 95% CI = −.27, −.01).

The specific indirect effects, each for 1 independent variable and mediator, are shown in Table 3. Results show that higher perceived severity of the effects on the adult child was associated with higher problem-focused and emotion-focused coping, which in turn were associated with higher PTG. Lower perceived self-control and treatment control was associated with lower problem-focused coping, which in turn was associated with lower PTG. That is, higher perceived self-control and treatment control was associated with higher problem-focused coping, which in turn was associated with higher PTG.

**Table 3.** Specific indirect effects for PTG, with perceived severity of effects on the adult child, perceived low self-control and treatment control, and the coping strategies (*N* = 109)

Predictor	Mediator	Dependent variable	Effect (*SE*)	*p*	95% CI
Perceived severity of effects on the adult child	Problem-focused coping	PTG	.14 (.06)	**.006**	.04, .26
Perceived severity of effects on the adult child	Emotion-focused coping	PTG	.12 (.05)	**<.001**	.04, .23
Perceived low self-control and treatment control	Problem-focused coping	PTG	−.10 (.05)	**.039**	−.23, −.01
Perceived low self-control and treatment control	Emotion-focused coping	PTG	−.03 (.03)	.321	−.10, .03

## Discussion

The aim of the current study was to investigate the associations between disease characteristics, illness representations, coping strategies, and PTG in young adults with parents diagnosed with cancer. In this research we implemented a structured model based on the SRM to understand the complex interplay of these factors.

The moderate PTG score that we found among young adults with parents diagnosed with cancer reveals diverse coping mechanisms and resilience levels. Previous research has indicated that facing crises can lead to both negative consequences, such as heightened emotional distress, and positive outcomes, such as personal growth (Henson et al. 2021; Liu et al. 2020a). Dealing with a parent’s cancer presents challenges but also offers growth opportunities (He et al. 2022; Morris et al. 2020). This experience allows individuals to grasp life’s fragility, value social connections, reassess priorities, and see opportunities differently (Liu et al. 2020b; Tedeschi et al. 2018). Consequently, the current study supports the idea of growth amid crisis for adult children coping with their parents’ illness, contributing insights to the research on crisis-induced growth in this context.

In this study, adult children perceived their parents’ cancer as significantly impactful, with a notable emphasis on the severe effects it had on them. Such a perception implies the profound influence that the parent’s cancer had on various facets of the participants’ lives, such as unmet needs and psychosocial difficulties (Landi et al. 2022). The reported low levels of perceived self-control and treatment control suggest a potential sense of powerlessness and limited influence over the trajectory of the illness, adding complexity to the challenges of coping with a parent’s cancer (Landi et al. 2022; Morley et al. 2016). Despite the perceived severity, however, the moderate level of problem-focused coping suggests a balanced engagement with practical challenges, indicating the young adults’ recognition of certain constraints in their ability to exert control. Concurrently, the moderate-low levels of emotion-focused coping indicate a measured approach to handling the emotional aspects of their parent’s illness, reflecting emotional resilience or adaptability (Morris et al. 2020). This combination underscores the intricate coping strategies employed by these adult children, emphasizing the necessity for a multifaceted and individualized approach to navigate the challenges associated with a parent’s cancer.

Our study’s findings bolster the assumptions of the SRM (Leventhal et al. 1980) by revealing significant associations between cancer illness representations, coping, and PTG. Even with the inclusion of a disease characteristic (disease stage), our results indicate that illness representations and coping strategies account for the variance in PTG. This finding suggests that subjective perceptions of cancer, as opposed to the objective characteristics of the disease, play a pivotal role in shaping the experience of PTG, and stands in line with findings from previous studies conducted in other disease contexts such as asthma (Shinan-Altman and Katzav 2021) and rheumatoid arthritis (Shinan-Altman and Afuta-Goldstein 2020). In essence, the way in which individuals perceive and interpret the impact of cancer on their lives seems to be a key determinant of their potential for growth in the aftermath of such a significant life stressor.

Our research highlights the role of coping strategies in mediating the association between illness representations and PTG in young adults with parents diagnosed with cancer. In line with the SRM, we found that the way adult children perceived the severity of their parent’s cancer significantly influenced their coping methods and subsequently their PTG. Coping is a behavioral strategy and an important mediating factor to relieve psychological stress (Nik Jaafar et al. 2021; Shao et al. 2022). For the young adults with parents diagnosed with cancer in the current study, perceiving the disease as more severe seemed to lead to a more intensive use of both problem-focused and emotion-focused coping strategies, enhancing PTG. On the other hand, lower perceived control over the illness seemed to result in less use of problem-focused coping and consequently lower PTG.

### Study limitations

This study has several limitations. We specifically targeted adult children between the ages of 20 and 35, given the specific features of this emerging adulthood cohort; as such, the findings may lack generalizability. Additionally, the potential impact of cultural factors on PTG may limit the applicability of our findings to other cultural contexts. Going forward, the age range should be expanded, and cultural factors should be considered to better understand age-related differences in coping and growth across diverse populations. In addition, the cross-sectional design precludes causal inferences, indicating a need for longitudinal studies so that changes over time in PTG can be observed. Furthermore, reliance on self-reported measures can introduce response biases; future studies could incorporate objective assessments or a mixed-methods approach for a more comprehensive understanding. Additionally, recruitment methods may have introduced bias, affecting generalizability, as participants who engaged might differ from those who did not. Future research should use more diverse recruitment strategies to minimize bias and improve generalizability. In addition, the oversampling of women, especially considering that women tend to report significantly more PTG than men, may skew the results, and future studies should aim for a more balanced gender representation. Furthermore, data on the time elapsed since the parent’s cancer diagnosis were not collected. Future studies should include this variable to better understand its impact on PTG. In addition, the lack of a control group or comparison to normative data limits our ability to determine if the observed PTG levels are unique to this population. Finally, by focusing on specific emotional and psychological impacts, other relevant aspects may have been overlooked. In future studies, broader impacts should be explored, including social and economic factors, to provide a more holistic view of the experiences of young adults with parents diagnosed with cancer.

### Clinical implications

The findings from this study underscore the necessity for tailored psychosocial interventions aimed at enhancing adaptive illness representations and effective coping strategies among young adults with parents diagnosed with cancer, specifically those aged 20–35. Clinicians should focus on helping these individuals develop positive illness representations, such as a sense of control and understanding of the disease, to foster PTG. Incorporating cognitive-behavioral techniques to modify maladaptive beliefs and promote effective coping mechanisms can significantly improve psychological well-being. This age group faces unique challenges, such as balancing personal relationships, career responsibilities, and financial independence, while providing care and support to their ill parent. Therefore, interventions should be personalized to address these emotional and practical challenges, promoting resilience and growth amidst adversity.

### Conclusions

This study illuminates the significant role of the SRM in understanding PTG among young adults with parents diagnosed with cancer. The findings reveal a substantial connection between the perceptions of illness severity and controllability, coping strategies employed, and PTG. Highlighting the necessity for tailored psychosocial interventions, this research underscores the importance of addressing individual perceptions and emotional responses to a parent’s cancer. The study offers practical implications for enhancing coping mechanisms and fostering resilience in this unique demographic. This contribution is pivotal in enriching the theoretical framework of PTG and improving support strategies for young adults with parents diagnosed with cancer.

## Data Availability

The data that support the findings of this study are available from the authors upon reasonable request.
